# Protocol for a randomised controlled trial to evaluate the effectiveness of improving tuberculosis patients’ treatment adherence via electronic monitors and an app versus usual care in Tibet

**DOI:** 10.1186/s13063-019-3364-x

**Published:** 2019-05-16

**Authors:** Xiaolin Wei, Joseph Paul Hicks, Pande Pasang, Zhitong Zhang, Victoria Haldane, Xiaoqiu Liu, Tingting Yin, Lixia Wang, Dachun Shi, Shiliang Ge, John Walley, Ross Upshur, Jun Hu

**Affiliations:** 1 Dalla Lana School of Public Health, University of Toronto, Toronto, ON Canada; 2 Nuffield Centre for International Health and Development, University of Leeds, Leeds, UK; 3 Shigatse Centre for Disease Control and Prevention, 7 Keji Road, Samzhubze District, Shigatse, Tibet China; 4 China National Centre for Tuberculosis Prevention and Control, China National Centre for Disease Control and Prevention, Beijing, China; 5 Weifang Medical College, Weifang, Shandong China

**Keywords:** Tuberculosis, Treatment adherence, Mhealth, Randomised controlled trial, Tibet

## Abstract

**Background:**

Treatment non-adherence is a serious challenge to effective tuberculosis (TB) control in Tibet. In this study we will pilot and evaluate the effectiveness of using new electronic monitors (e-monitors) and a smartphone app to improve treatment adherence among new pulmonary TB patients in Tibet.

**Methods:**

We will use a multicentre, parallel-group, individually randomised controlled, superiority trial with blinded outcome evaluation and unblinded treatment. We will randomise new pulmonary TB outpatients (aged ≥ 15 years old and free from communication impairment) from Shigatse, Tibet to either the intervention or control arm in a 1:1 ratio at the time of their diagnosis. All patients will be treated according to the World Health Organisation standard 6-month TB treatment regimen and the China National TB programme guidelines. Intervention arm patients will be given their medication via e-monitors that have automatic voice reminders, and record medication adherence data and share it with health staff via Cloud connection. Intervention patients will also be encouraged to receive smartphone-based video-observed treatment if their adherence is problematic. Control arm patients will receive their medication in e-monitors that will collect medication adherence history, but will have their reminder function deactivated and are not linked to the app. The primary outcome is the rate of poor adherence, measured monthly during treatment as a binary indicator where poor adherence means missing ≥ 20% of doses in a month. We will conduct a qualitative process evaluation to explore operational questions regarding acceptability, cultural appropriateness and burden of technology use, as well as a cost-effectiveness analysis and an analysis of the long-term effects of the intervention on TB control.

**Discussion:**

Our study is one of the first trials to evaluate the use of e-monitors and smartphone apps for customised treatment support in low- and middle-income countries (LMICs). All intervention activities are designed to be embedded into routine TB care with strong local ownership. Through the trial we intend to understand the feasibility of our intervention, its effectiveness, its cost-effectiveness and its long-term impacts to inform future scale-up in remote areas of China and other LMICs.

**Trial registration:**

Current Controlled Trials, ID: ISRCTN52132803. Registered on 9 November 2018.

**Electronic supplementary material:**

The online version of this article (10.1186/s13063-019-3364-x) contains supplementary material, which is available to authorized users.

## Background

Treatment non-adherence is of paramount challenge in tuberculosis (TB) control, and an important driver of the emergence of TB drug resistance in Tibet, due to its sparse population density, severe weather conditions, long travel distances and shortage of human resources to enable implementation of directly observed treatment (DOT). Similar problems have been identified in other parts of China [1]. Patients either self-administer their treatment or receive inadequate supervision from a health worker, normally the village physician [2, 3]. This has resulted in poor treatment outcomes and high default rates. Therefore, the Tibetan TB programme urgently requires applicable and affordable alternatives to improve treatment adherence.

Digital technology provides a promising tool to improve treatment adherence. Existing literature suggests that electronic reminders and medication packaging interventions, such as Short Message Service (SMS) reminders, electronic device reminders and pager reminders, markedly improve short-term adherence for patients with chronic diseases [4, 5]. Typical characteristics of these mhealth technologies include recording of dosing events, storage of records, audiovisual reminders, digital displays, real-time monitoring and performance feedback. A trial in Ghana demonstrated that web-based SMS reminders substantially improved patient adherence to diabetic care and their clinical outcomes [6]. Recent studies have included electronic monitors (e-monitors) as they deliver detailed, precise, and objective data on daily adherence within a real-world setting [7–10]. However, no studies have shown a long-term effect for patients with chronic diseases [4, 5, 11, 12]. This research gap is particularly apparent for HIV patients undergoing chronic antiretroviral therapy [13, 14]. As such, the existing literature highlights the need to tailor intervention delivery to meet versatile patient demands. That is, the new technologies need to interact beyond the patient interface to include multi-media technologies, integrating models of patient care delivery and connecting patients with healthcare professionals [11, 13, 14].

In TB care, e-monitors and automated reminders have emerged to improve the quality of care delivery with accurate, real-time, detailed dosing information, and dedicated communication channels between patients and healthcare workers to discuss their concerns [15]. Real-time treatment adherence can support healthcare workers to identify patients who need help and focussed support, rather than having to spread their efforts around all patients. In addition, new apps, such as WeChat, can transmit texts, photos, audio and videos in real-time, and have become popular among Tibetans. This, therefore, provides the possibility of providing video-observed treatment (VOT), an alternative to direct observation using real-time or recorded videos for patients, based on patient treatment histories. Despite the great potential, there is limited evidence supporting the use of such new technologies to support TB management. To our knowledge, worldwide there has only been one randomised controlled trial (RCT) that assessed using e-reminders to improve TB adherence, but this study (based in the Chinese provinces of Heilongjiang, Jiangsu, Hunan, and Chongqing) used an older e-monitor that could not connect with smartphone apps enabling VOT [16].

After piloting our intervention and study processes for feasibility we will evaluate, via a RCT in two districts of Shigatse in Tibet, whether using medication e-monitors with Cloud connections improves the adherence of new pulmonary TB patients to their medication, compared to using inactivated e-monitors. The activated e-monitors will provide patients with automated voice reminders to take their medication, and by linking to a smartphone app will enable patients to receive treatment education, and will enable clinicians to access real-time patient medication adherence data, and will enable real-time patient-clinician communication, including VOT if necessary. Monitors in the control arm will not be activated for voice reminders and not connected to smartphone apps. We will also explore implementation questions regarding acceptability, cost-effectiveness and long-term effects to inform future scale-up in remote areas of China and other low- and middle-income countries (LMICs).

## Methods

This protocol is reported according to the Standard Protocol Items: Recommendations for Interventional Trials (SPIRIT) guidelines (see Additional file 1 for details) [17].

### Study design

We will use a multicentre, parallel-group, individually randomised controlled trial with one intervention and one control arm, using a 1:1 allocation ratio, to evaluate whether the intervention is superior to the control treatment. The study design is informed by the Medical Research Council framework [18] on complex interventions and implementation science principles [19] with an embedded theory-based process evaluation [20] to examine operational questions regarding acceptability, cultural appropriateness, and burden of technology use. In addition, we will conduct an incremental cost-effectiveness analysis to inform future scale-up. We also plan to do a follow-up study to compare the intervention’s impact on TB relapse rates after patients finish their treatment.

### Setting

We will implement the study in two districts of Shigatse Prefecture in China’s Tibetan Autonomous Region: one urban area called Samzhubze, and one rural area called Sa’gya. Shigatse Prefecture is located west of Lhasa with an average altitude of 4000 m above sea level. Population density in Shigatse is very low (4/km^2^). The prefecture covers an area of 182,000 km^2^ of harsh and rugged terrain with paved roads connecting county centres. Over 90% of its 800,000 residents are ethnic Tibetans. The 2014 TB survey revealed that the prevalence rate of pulmonary TB was 758/100,000 (0.76%), almost twice China’s national average [21, 22].

The two project sites currently face challenges to provide standard DOT to ensure TB treatment adherence. TB care is only available from public health providers including: (1) TB dispensaries located in Centre for Disease Control (CDC) facilities for clinical care and general coordination, (2) township hospital (primary care facilities) physicians for home visits and supervision of village physicians, and (3) village physicians, often as treatment supervisors. According to China’s National Tuberculosis Programme (NTP) guidelines, new pulmonary TB patients should be treated in the community and should visit the district TB dispensary every month to refill their medicines. However, the serious shortage of health staff, plus the long travel distances and severe weather conditions make regular home visits often impossible. In practice, patients typically receive self-administered therapy (SAT) with limited calls/visits from health staff, and loss to follow-up rates have been very high. Most patients visit the TB dispensary once every 2 months, if not longer. Across Shigatse in 2016, only 72% (769/1073) of new pulmonary TB cases completed treatment. Among patients who did not complete treatment 83% (252/304) were lost to follow-up. Treatment completion rates vary greatly by district. In our two study districts a total of 269 patients were registered in 2016, but only 38% (102) completed treatment, while 60% (162) were lost to follow-up and 1% (3) died. Many patients, mostly poor farmers, were lost to follow-up during the intensive phase as they received little education and support from health workers due to a lack of communication.

### Eligibility and recruitment

In each district, patients with presumed TB and newly confirmed pulmonary TB will be referred to the TB dispensaries by health workers from other public and private health facilities. Individuals can also present themselves if suspecting having TB. All patients will be diagnosed in the TB dispensary according to national and international guidelines of TB care [23]. Eligible TB patients are those: (1) aged ≥ 15 years, (2) who are starting on standard 6-month short-course chemotherapy on an outpatient basis, (3) are free from any communication impairment (mental, visual, auditory or speech), and (4) do not have any family members within the same household who have already been enrolled into the trial. We will recruit patients into the study via TB physicians in the TB dispensaries who will screen patients for eligibility, and explain the study purpose and obtain informed consent. The TB physicians also act as TB coordinators responsible for reporting data to the NTP. Based on existing routine TB data across the two districts we expect to recruit 300 new pulmonary TB patients in 15 months. We will maximise patient enrolment through fully informed consultations provided by TB physicians.

### Randomisation and blinding

Following recruitment, patients will be randomised to either the intervention or the control arm in a near 1:1 ratio, using a computer-generated randomised permuted block design. The design will use random block lengths of 2, 4 or 6, and include an initial unbalanced block, with a maximum of one additional patient in either arm, to further hide allocation sequences, which means that the ultimate sample size and allocation ratio will vary very slightly from what is planned dependent on the unbalanced block. The allocation will be stratified by district, with one district having approximately 160 patients and another having approximately 140 patients, with a near equal allocation ratio in each. JPH will generate the allocation sequence, and will have no further role in the randomisation or allocation process. The allocation sequence will be used by the study team to print individually numbered allocation cards, which will be placed in sequentially numbered, sealed, opaque envelopes before being delivered to study sites. The TB physicians will allocate each recruited patient after their enrolment by opening the correct envelope in the sequence, and will provide the e-monitor setup according to the patient’s allocation. Research team members will educate the TB physicians on the importance of following the correct sequence, and local CDC staff will make monthly visits to the TB dispensaries to carry out spot checks and ensure the randomisation procedures are being correctly followed. It will not be possible to blind providers or patients due to the nature of the intervention. However, we will take measures to ensure a blinded outcome evaluation using: (1) the ‘PROBE’ design of blinded outcome evaluation [24], and (2) blinding those analysing the data to treatment status.

### Treatment processes: all arms

All patients will be treated according to the standard WHO DOTS programme and the China NTP guidelines using isoniazid, rifampin, ethambutol and pyrazinamide for 2 months (3 months for sputum-smear-positive patients whose sputum smears have not converted to negative at the end of 2 months), followed by isoniazid and rifampin for 4 months, under daily fix-dose combination (FDC) for the entire treatment course [23]. According to the NTP guidelines, patients will be given the choice of SAT, or treatment with a supervisor, mostly village physicians but sometime a township hospital health worker. The health workers who act as treatment supervisors will receive RMB 60 (US$10) from the government when completing 6-month support to a TB patient. The township hospital health workers also have the role of visiting patients’ homes, and supervising village physicians. In the two districts patients should visit the TB dispensary at least every 2 months to meet with their TB physician and refill their FDC medications using the e-monitor box, which will collect data on the number of medications unused and refilled each time they are opened. When patients meet with the TB physician they will also count any leftover medicines. E-monitors will be returned to district TB dispensaries at the end of treatment and reused when possible.

### Intervention arm

**Fig. 1:**
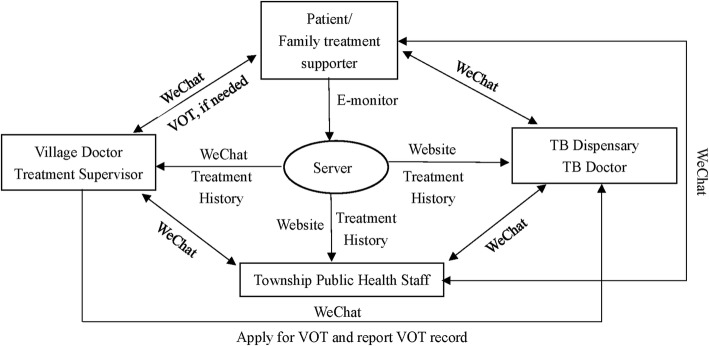
Data flow between patients, health providers, and the server using electronic monitors and the WeChat app

Figure 1 shows the framework of intervention strategies. The e-monitors in the intervention arm have two main functions beyond storing the TB medication. First, they will remind patients to take their medicine on time using human voice recordings. Second, they will transmit patients’ adherence history to a Cloud-based server, linking with computers and a smartphone app. At recruitment the TB physician will demonstrate how to use the e-monitor box and WeChat app. The treatment supervisor will visit the patient’s home within the first week of treatment, and solve any remaining problems. During recruitment or at the first home visit by the treatment supervisor, a family member will be chosen to act as a treatment supporter. The family member must live in the same house as the patient, care about the patient, and be literate in using the WeChat app. Then, patients or their family members (if the patients are not able to use a smartphone or WeChat) will be invited to setup the WeChat app on their smartphones, and connect with their TB physician and treatment supervisor. When appointed, the family member will be trained by the TB physician and the treatment supervisor about their responsibilities for providing psychological support to patients and facilitating patients use of the e-monitor and the WeChat app. TB physicians and treatment supervisors will add the patient or the family member into their WeChat. Patients, treatment supporters, supervisors and physicians will be able to have direct but distinct communications. In addition, patients will receive audio/video-based health education messages through WeChat sent by the treatment supervisor and TB physician.

Using the WeChat app and also their computers connected with e-monitors through the Cloud, health workers will be able to monitor patient adherence history and outpatient visits. Patients who report consistently taking their medications will continue SAT. TB treatment supervisors or TB physicians will identify patients at high risk of being lost to follow-up based on whether the patient: (1) expresses serious concerns about maintaining their adherence, (2) skips three consecutive doses or (3) refuses to start or continue treatment. Treatment supervisors will be trained that if their patient is considered a high risk of becoming lost to follow-up they should explore with the patient their reasons for non-compliance and what help may be needed. The treatment supervisor or TB physician will then try to initiate VOT while providing any help needed. The treatment supervisor will document the start and end time of VOT. Patients who accept the invitation to begin VOT will be given instructions either in the TB dispensary or at home during the visit of their treatment supervisor. Using the WeChat app VOT can be done with the TB supervisor via a live video conversation or via recorded video/pictures showing them taking their medicines. VOT will be provided until three consecutive doses are completed on time, then the patient will switch back to SAT using their e-monitors. Patients who do not agree to VOT will be called daily by treatment supervisors until three consecutive doses are completed. We anticipate that less than 20% of TB patients will need VOT, and mostly during the initial phase or at the end of the treatment.

TB physicians, township public health staff and village physicians will receive training on a revised operational NTP guideline that incorporates using the e-monitor box, WeChat, and VOT based on our previous experience [3], as well as how to communicate using the tools.

### Control arm

The control arm will practice usual care except that patients will use deactivated e-monitors without automated voice reminders to only collect treatment adherence data. The data will be encrypted on the Cloud and will thereby not be available to health workers during the study. At each visit to the TB dispensary, patients will refill their medications in their e-monitor boxes every 2 months. Treatment supervisors are advised to visit patients at least once a week according to the NTP guideline, but this will be at their own discretion. We will not invite patients to connect with their TB physician or treatment supervisors through the WeChat app. Treatment supervisors will contact patients through traditional means, such as physical visits or phone calls. We will not select family treatment supporters.

### Outcomes

The primary outcome will be the rate of poor adherence measured per month across the 6/7 months of the standard WHO DOTS programme for new pulmonary TB patients. We will calculate the outcome from monthly level data for each patient indicating the number of doses missed per month, with poor adherence in a given month defined as the patient having missed ≥ 20% of doses in that month (equivalent to missing ≥ 6 out of 30 doses in a given month). This threshold has been commonly used in other disease areas [25] and in a similar trial as a relevant indicator of treatment adherence [16]. Our secondary outcomes are: (1) the patient-level percentage of total doses missed over the 6/7 months of treatment (calculated for each patient based on the total number of doses missed out of the total possible number of doses), and (2) a patient-level binary indicator of overall poor adherence (defined as ≥10% of total doses missed, which is the NTP definition of non-adherence). Our remaining secondary outcomes will be based on World Health Organisation (WHO) standard definitions of TB treatment outcomes [26]: (3) treatment completion rate (‘a TB patient who completed treatment without evidence of failure but with no record to show that sputum smear or culture results in the last month of treatment and on at least one previous occasion were negative, either because tests were not done or because results are unavailable’), (4) loss to follow-up rate (‘a TB patient who did not start treatment or whose treatment was interrupted for two consecutive months or more’), (5) poor treatment outcome rate (defined as death, treatment failure (‘a TB patient whose sputum smear or culture is positive at month 5 or later during treatment’) or patient loss to follow-up), and (6) sputum conversion rate at the end of the second month [27].

### Sample size

As there are limited analytical sample size approaches available for longitudinal outcomes we used a simulation approach. We based our subsequent power calculation on our primary outcome of monthly poor adherence, and assumed a 40% monthly poor-adherence rate for the control arm [16]. For our simulations we created six binary poor-adherence outcomes (i.e. one per treatment month) for patients, varying the total number of patients between simulations to explore power, but always assuming a 1:1 treatment arm allocation ratio. The outcomes were randomly generated from a Bernoulli distribution with a fixed probability of success (i.e. probability of monthly poor adherence), which was always set to 0.4 for the control arm, but was varied between simulations for the intervention arm. In addition, in each simulation all patients’ outcomes were generated with a fixed level of correlation between their monthly outcomes, based on an exchangeable correlation structure. These outcome data were generated using the R package simstudy [28]*,* based on the Emrich and Piedmonte algorithm [29]. We explored the effect on power of assuming an AR1 correlation structure instead, but as our simulations showed that this resulted in less conservative estimates of power, and because we had no similar data to explore the robustness of this assumption and no strong reason to assume an AR1 structure, we assumed an exchangeable structure instead. After generating the simulated data we then used the R package geepack [30] to fit a generalised estimating equation (GEE) to the data with binomial errors and an identity link, which allowed estimation of the absolute difference in treatment arm outcome proportions (see the ‘Statistical analysis’ section for more details) [31]. The GEE included a covariate for treatment arm and a categorical variable for month. We then extracted the Wald-based two-sided *p* value associated with the coefficient for the treatment effect estimate. Finally, for each set of parameter assumptions we repeated this process 1000 times and calculated the resulting power based on the proportion of *p* values ≤ 0.05. We assumed no missing data due to patients who become lost to follow-up still being able to contribute 100% of their outcomes in the study. Assuming an expected monthly poor-adherence rate of 40% in the control arm and a moderate within-patient correlation between monthly poor-adherence outcomes of 0.5, we require 300 patients to detect an absolute reduction of 12 percentage points in the intervention arm outcome (i.e. an intervention-arm monthly poor-adherence rate of 28%) with 81.5% power.

### Pilot stage

We will assess the feasibility of implementing the intervention and running our trial processes through a pilot study that will precede the main trial. Specifically, during the preparation period we will invite four new pulmonary TB patients (two in each district) to be randomised into either the intervention or control arm as pilot cases, allowing us to test the randomisation and recruitment processes. We will then follow them up for 2 weeks to assess the feasibility and acceptability of: (1) using the e-monitor boxes for medication storage, (2) using the Cloud-based server for monitoring adherence history, and (3) using the WeChat app for patient-health worker communication. We will also pilot test the feasibility of using VOT in the two patients who are allocated to the intervention. We will revise the implementation plan based on our experiences from the pilot.

### Process evaluation

**Fig. 2:**
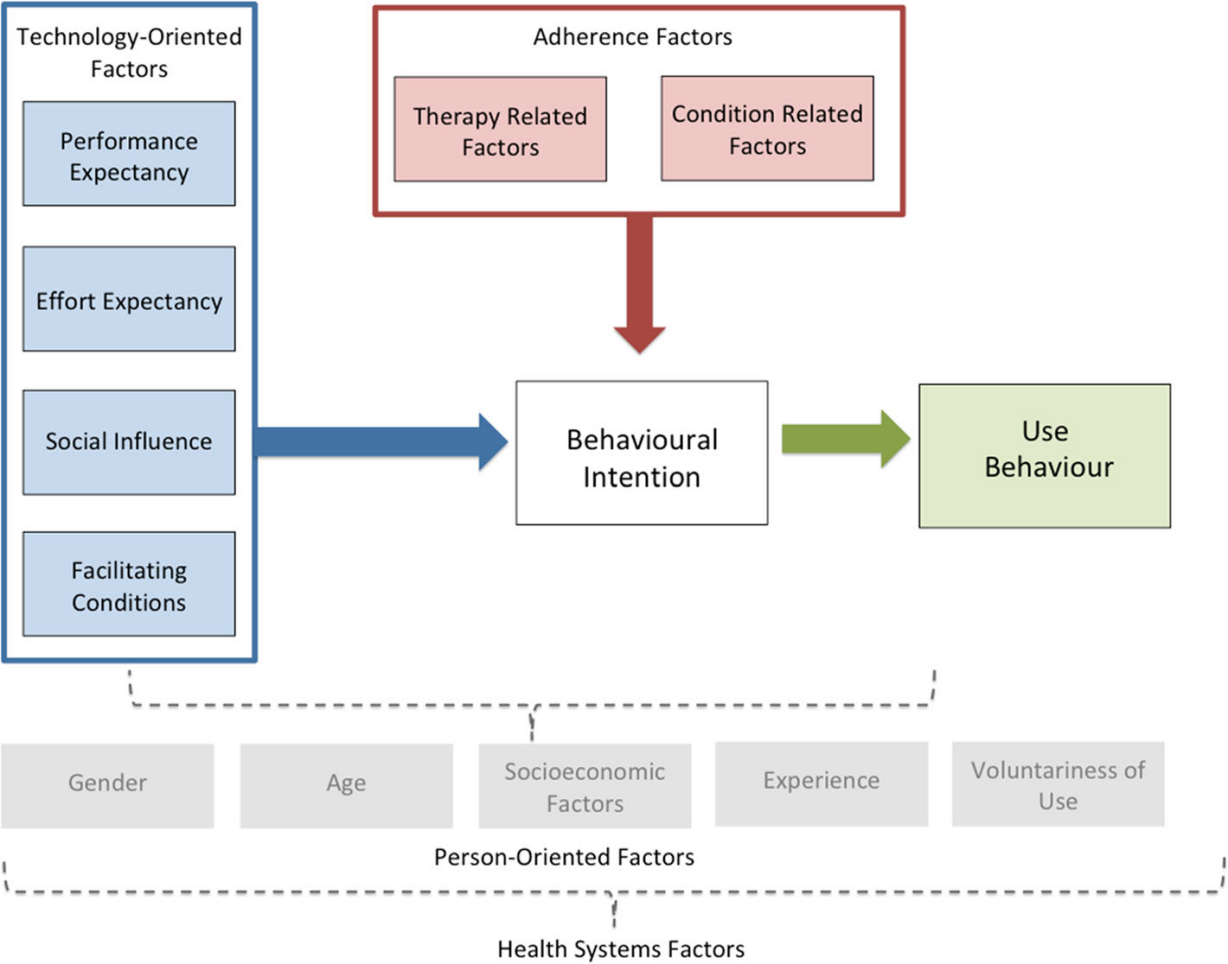
Theoretical framework to examine technology acceptance and use for adherence factors

Theoretical framework: we will employ an adapted version of the Unified Theory of Acceptance and Use of Technology (UTAUT) model [32, 33] to guide our process evaluation to assess issues that influence patients’ behaviour change in relation to both the technology used, such as acceptance, feasibility, appropriateness, adaptability, as well as adherence behaviours (Fig. 2). The UTAUT has been widely used to understand information technology adoption in general. The adapted UTAUT model has four constructs through which to explore technology-oriented factors that shed light on user experiences interacting with a given technology. These factors are: (1) performance expectancy, (2) effort expectancy, (3) social influence, and (4) facilitating conditions. Performance expectancy includes the perceived usefulness and personal outcome expectations associated with technology use. Effort expectancy is the perceived ease of use and complexity of the technology. Social influence includes subjective norms and technology use within a user’s social context. Facilitating conditions include perceived behavioural control and wider contextual circumstances that support the use of technology. Together these factors provide a comprehensive understanding of the patient’s experience with the technology.

We also included adherence relevant aspects of the WHO adherence framework into our theoretical framework [34]. Condition-related factors are the illness-related demands faced by the patient which ultimately impact the patients’ risk perception, treatment beliefs and the priority that they place on adherence. While therapy-related factors include side effects, treatment duration, treatment failures and experience of side effects [34]. These indicators may either motivate or dissuade patients from adhering to treatment plans. Though our intervention may satisfy technology-oriented needs of a patient, their treatment plan and their lived experience may pose unforeseen challenges to their behavioural intention and change. All the above factors will be considered with contextual and person-oriented factors including gender, age, socioeconomic factors, experience and voluntariness of technology use.

Our process evaluation is designed to explore factors highlighted by the modified UTAUT model from the perspective of patients, treatment supervisors, and physicians. We will record the e-monitor’s defects, connectivity, and the operation within existing health and logistics conditions through programme logbooks. We will also record each supervision trip. We will report the proportion of patients having a smartphone, able to enrol in, and their technology use. We will measure the accuracy of patient adherence history collected by e-monitors against anti-TB drug refills/ counting in TB dispensaries at each time of patient refills. District TB physicians will record the persistence of using e-monitors over 6/7 months, the proportion of patients who need VOT, start VOT, and the persistence of VOT (over 3 days until satisfactory adherence).

We will conduct semi-structured in-depth interviews to understand acceptability, cultural appropriateness, and burden of technology use. We will recruit and interview 24 patients (18 from the intervention arm and 6 from the control arm), 12 family treatment supporters, at least 12 treatment supervisors, six township treatment supervisors and four TB physicians. Among patients in the intervention arm, we aim to explore differences between patients who demonstrate adherence and those who do not. We also aim to explore differences in technology uptake, adoption and sustained use between patients and identify whether there are different stages of uptake and adoption. Questions will explore smartphone literacy, experience and usability of the new technology, overall satisfaction, and any concerns (e.g. understanding, learning experience, time consumption, communication quality, confidentiality, data-usage costs). Due to culture norms, we will pay specific attention to women regarding confidentiality/privacy issues in communications using WeChat and the VOT.

### Economic study

We will conduct an incremental cost-effectiveness study that will collect costs of the implementation package including staff, equipment, and supplies – ‘incremental’ to those required in usual (control arm) care. Overall incremental cost-effectiveness ratios for the service intervention and health outcomes, based on our primary and secondary endpoint of treatment completion rate, will be computed where possible. Simple unit costs of implementing the intervention including cost per district and per patient will be computed. The total per patient cost will be calculated as the sum of three elements: (1) the cost of clinic consultation estimated with physician’s time input and unit salary; (2) the cost of patient follow-up measured with frequency of home visit/using WeChat, average time input for the two approaches and their unit salaries, plus travel cost where applicable; and (3) medication cost including inpatient and outpatient health service charge, and indirect cost (travel, caregiver cost, etc.) when a patient is hospitalised. The unit implementation cost will be calculated as the sum of: (1) cost of staff time of both trainers and trainees; and (2) preparation of intervention materials including e-monitors and printings. Using published data, as available, we will estimate the benefits to: human health including potentially reduced multiple-drug resistance TB or TB death, and productivity gains.

### Follow-up study

We plan to follow up all patients using their national ID numbers which will be recorded in the NTP reporting system as a routine practice in Tibet. Follow-up will start from when a participant completes their treatment with any treatment outcomes including loss to follow-up. We will track any relapse cases reported in Tibet using the national TB reporting systems within 12 months of their treatment completion. Some patients may travel to other provinces to work where national ID number reporting is not mandatory for TB cases. To mitigate this gap, we will conduct a short survey regarding their TB status and the place of treatment (if any) by the end of the follow-up study through their treatment supervisors. We will also collect all mortalities using the vital registration system in China where the national ID has to be input. The primary endpoint of the follow-up study is TB relapse and the secondary endpoint is mortality. We will evaluate whether these outcomes differ between the intervention and control arms using the same methods of analysis as outlined below for the main trial indicators. However, where patient follow-up data cannot be collected we will conduct complete-case analyses by excluding those patients with missing data. This study will be reported separately.

### Ethical approval

The trial protocol has obtained ethical approval from the Office of Research Ethics at the University of Toronto (Ref: 36569) and the Ethics Review Committee of the Tibet Centre for Disease Control and Prevention (Ref: 006).

### Data collection

In addition to the requirement by the China NTP, all health staff in this study will have to sign an agreement to keep all patient information confidential and only use it for the purpose of TB care. All patient information stored in the WeChat app at the provider’s end will be deleted immediately once the patient has completed treatment or been lost to follow-up. Patient information, including name, age, sex, address, education, profession, diagnosis, and treatment outcomes, will be routinely collected by the NTP. We will also collect routinely recorded treatment outcomes data from the two districts, including conversion at the second month, loss to follow-up, death, and sputum smear results at the fifth and sixth months. We will collect all these data during monthly monitoring visits in the two districts. We will use double entry and check a random subset of the data to ensure data quality. We will use these data to calculate our secondary indicators. The interventions are unlikely to increase potential risks to patients. However, any adverse events of the interventions will be reported to the research coordinators in the Shigatse CDC by TB physicians within 24 h and will be recorded in work logs.

For this study we will allocate a unique participant number for each patient in our dataset to mask patient identifiable information such as ID, names, and addresses. Research staff will only have access to the participant number and non-identifying information such as treatment outcomes. All the patients’ national ID numbers will be recorded in the TB reporting system as routinely required, which will be used to track any relapse cases 12 months after the patient has completed their treatment.

We will measure the adherence indicators based on data collected by the e-monitor in both intervention and control arms, which will be downloaded from the Cloud-based database. To ensure that all missed doses are accurately recorded we will cross-check the adherence history recorded from e-monitors with pill counts manually recorded at the end of each outpatient visit, and we will record as the true value whichever is the larger number of missed doses from the two data sources. The TB physician will also conduct a short questionnaire survey asking: (1) if patients have had access to and/or used the WeChat and e-monitors, (2) how much time patients have spent using the e-monitors and WeChat, and (3) any medical costs. We will also conduct a questionnaire for physicians and treatment supervisors at the end of the trial period to measure their time cost in supporting patient treatment. We will collect implementation costs using researchers’ work logs, including the number of trainees, trainers’ labour and travel costs, and the cost of training materials and the e-monitor boxes. We will also record and audio tape the interviews with patients and health staff for the process evaluation. Information from the interviews will only be accessed by research staff, and anonymised during analysis.

### Statistical analysis

Our primary analysis population will be the ‘intention-to-treat’ population, which will include all randomised patients analysed according to their original treatment allocation, irrespective of their subsequent adherence or how they were actually treated. The Consolidated Standards of Reporting Trials (CONSORT) guidelines for reporting parallel-group randomised trials recommend presenting absolute measures of effect for binary outcomes due to their increased relevance for practice and policy, as well as relative measures of effect. Generalised estimating equations (GEEs) have been shown to produce robust estimates of the absolute difference in treatment effect for binary outcomes from multi-level trial data [31]. Therefore, we will analyse our monthly binary primary outcome of poor adherence using a GEE, with binomial errors and an identity link, to estimate the absolute difference in the proportion of poor adherence across all treatment months between the intervention and the control arms (intervention minus control). We will assume an exchangeable correlation structure for the repeated outcomes within individuals, and estimate parameter standard errors as ‘Huber-White’ or ‘robust’ standard errors. In this analysis we will also adjust for effects (covariates) of month (as a categorical variable), stratum, and a range of sociodemographic characteristics: age (categorical: 15–49, 50 + years), sex, marriage status (married, never married/not currently married), and employment status (employed: farmer, employed: other, unemployed/retired). We will base our principle inference about the effectiveness of the intervention on this main analysis. We will also repeat this analysis but excluding all covariates other than month and stratum as a sensitivity analysis to explore how robust the treatment effect is when not adjusting for those potentially imbalanced covariates. To explore how the treatment effect differs between treatment months we will also repeat our main analysis but also include an interaction between treatment group and month (as a categorical variable) in the model, while also only including stratum as an additional covariate because our parameter to sample size ratio will be constrained. Because the approach of using an identity link in a GEE with binomial errors is not yet widely used, and to facilitate meta-analyses of odds ratios (which are more commonly estimated) we will also repeat the main analysis but using a GEE with binomial errors and a logit link to estimate the odds ratio for the odds of poor adherence in the intervention arm compared to the control arm (based on taking the exponential of the treatment effect coefficient). We do not expect any missing data because we are only collecting basic covariate data at baseline, and if patients are lost to follow-up at any point then they will still contribute 100% of their primary outcome data because their adherence status would be validly recorded as non-adhering from the point at which they were lost to follow-up. However, if their primary outcome data are missing for an unexpected reason (e.g. loss of data) then we will ensure that the ‘intention-to-treat’ analysis population is maintained in all analyses by imputing the missing outcome data assuming the worst outcome of poor adherence for those months where data are missing.

For our secondary outcome of the patient-level percentage of total doses missed over 6 months of treatment, assuming the outcome is normally distributed we will analyse it using a linear regression model including the same covariates, other than month, as used in our primary outcome main analysis. If the data are substantially non-normally distributed we will apply a suitable transformation to the data to achieve normality. If any dose data are missing we will ensure that the ‘intention-to-treat’ analysis population is maintained by imputing the missing outcome data assuming the worst outcome, i.e. that the dose(s) were missed. For all remaining secondary outcomes, which are all binary, we will use a ‘logistic regression average-risk-difference’ analysis approach to obtain absolute measures of treatment effect. In these analyses we will again adjust for the same covariates, other than month, as used in the main primary outcome analysis. This approach has been shown to provide robust estimates of absolute treatment effects for RCTs with binary outcomes [35]. As with our primary outcome we will also conduct covariate-sensitivity analyses for all secondary outcomes where we will repeat the above analyses but without adjusting for any covariates other than stratum. For our secondary outcome of overall poor adherence if any dose data are missing we will follow the same approach as for our analysis of the patient-level percentage of total doses missed outcome. All patients should be assigned a WHO standard TB treatment outcomes. However, if any patients are missing these outcomes then we will assume the ‘worst’ outcome (e.g. for the treatment completed outcome we would assume they did not complete treatment).

Lastly, we will also conduct a small number of subgroup analyses to explore whether treatment effects vary between the subgroups. For our primary outcome we will use the same main analysis approach planned above (of using a binomial-identity GEE), but we will add an interaction between treatment group and the relevant subgroup identifier into the analysis to estimate the difference in treatment effect between the subgroups. We will conduct subgroup analyses for our primary outcome for the subgroups (1) sex, (2) age, and (3) travel time to the relevant district TB dispensary, and we will compare between men and women, less than the median age and greater or equal than the median age, and less than the median travel time and greater or equal than the median travel time. We will also conduct subgroup analyses for our secondary outcome of treatment completion for the same range of subgroups. To do this we will use a logistic regression model that includes covariates for treatment, subgroup, the interaction between treatment and subgroup, and the same range of covariates as noted in our main analysis except month.

We will claim statistical significance at the 5% level, and base our inferences on the two-sided *p* values and associated 95% confidence intervals of the treatment effect estimates. All outcomes will be analysed at the end of the trial, and no interim analyses are planned for this study.

### Analysis of process evaluation

Both quantitative and qualitative data will be analysed. Quantitative data will be summarised, described and analysed using appropriate statistical methods, including multiple linear regression for continuous outcomes and multiple logistic regression for binary outcomes. Qualitative data analysis will use a thematic approach to discover emergent themes. Notes will be reviewed after each interview to identify emergent topics and allow for exploration in subsequent interviews. Data will be translated and transcribed, then analysed using NVivo 10.

### Trial management

Prof Xiaolin Wei from the University of Toronto and Dr. Jun Hu from Shigatse CDC will be the co-guarantors of the trial who have full access to the trial dataset. An external-member-led data management committee (DMC) will be established to: (1) safeguard the safety and privacy of patients involved, and to (2) ensure that all data are collected according to agreed ethical guidelines, properly stored and only used for research purposes. We will also form an external-member-led trial steering committee (TSC), consisting of members from China NTP, Tibetan TB programme, key members of the trial team. Annual meetings will be held for both the DMC and TSC. Important protocol modifications will be discussed during the meetings.

## Discussion

Our study is one of the first trials to evaluate the use of e-monitors linked to a smartphone app to customise patient support in LMICs and increase adherence to treatment. The study fits well with the China NTP’s priority to promote new technologies to improve TB care in remote areas. Previous studies have revealed that only new technologies that improved communications with patients demonstrated benefits in improving disease control outcomes in programmes [11, 12]. In our trial, we will employ an e-monitor that has been piloted in other geographically challenging areas of China (e.g. Inner Mongolia and Xinjiang) and shown good usability. Instead of launching a new app, we will link patients with health worker through an existing app, WeChat, which is widely used among Tibetans. Secondly, we will actively involve patient family members who have good literacy in using WeChat, and are willing to provide support to the patient physically and psychologically. Thirdly, all intervention activities are designed to be embedded into the routine practice of TB care with strong local ownership. The project is co-led by Shigatse Centre for Disease Control and University of Toronto, with a trial unit established in Shigatse to coordinate trial activities. We will engage the TB programme at the national, provincial, and frontline levels in developing guidelines and training modules to ensure that the materials are ready to be adapted to other remote areas through the NTP. Fourthly, the trial is designed to fit into the cutting-edge implementation science principles that include a theory-based process evaluation and economic evaluation to inform scale-up in remote areas with similar challenges. Fifthly, we understand that adherence is a two-way interactive process between the patient and their physician(s). Our definition of the poor-adherence rate reflects part of the adherence dynamics, such as initiation, discontinuation, and implementation, that change on a monthly basis [36]. This indicator, as used in a previous similar trial, is more sensitive to measure patient behaviour change compared with the overall dose or adherence rate, and is more closely related to the objective of DOTS in maintaining sustained adherence throughout the 6/7 months of treatment [16].

There is a risk that patients may stop using e-monitors during their treatment. We will train treatment supervisors in both arms to check the status of e-monitors during their visit to patients. Patients will be asked to take their e-monitors to clinics for any medication refills. When patients must travel for a long time during their treatment, we will coordinate with TB physicians in their influx areas to ensure that patients continue to use the e-monitors.

Several other limitations and challenges need to be noted. First, we cannot blind the health providers or patients, which is very likely to introduce biases, particularly performance bias (for both health worker and patients) and/or the Hawthorne effect. However, we will minimise any risk of reporting bias by collecting patient adherence history objectively and prospectively through the e-monitors, which should prevent interference by health providers or patients. We will also ensure that health workers provide equal care to patients in both arms during the training. Second, many TB patients in Tibet are illiterate [22], but are able to use a smartphone and WeChat because: (1) contacts in WeChat can be recognised by icons, and (2) WeChat allows sending audio and video recording by smartphones rather than text alone. We will also employ a family member who has good literacy in using WeChat to assist the patient. Third, health providers, such as TB physicians and treatment supervisors, are very likely to deal with patients both from the intervention and control arms. To reduce the contamination, we will ensure that correct procedures are followed through our training and project supervision visits. Fourth, molecular tests, such as culture and drug susceptibility tests, are not available in Shigtase, while GeneXpert only has limited use because of required equipment shortages. Thus, we are not able to diagnose drug sensitive or resistance cases, although this does reflect the reality of the context and mean the trial is pragmatic in relation to diagnosis and case mix. Fifth, we will provide patients in the control arm with a deactivated e-monitor box which is different from routine practice. This may increase patients’ adherence to medications in the control arm, which is most likely to result in an estimate of the intervention’s effectiveness that is lower than if we were comparing it to existing practice without the use of e-monitor boxes. However, we have made conservative estimates of the expected rate of poor adherence and its difference between the two arms. Having the deactivated e-monitor boxes in the control arm also makes the trial less pragmatic. Sixth, at this stage we envisage that patients with problems with their hearing, viewing, and speech will be hard to be oriented using the new techniques in general, and that they should receive special support and care to complete the treatment regimens. We have, therefore, excluded them from the trial, but this obviously limits the generalisability of the results to those patients without these issues.

### Trial status and timelines

The trial was registered at Current Controlled Trials: ISRCTN52132803 on 9 November 2018 (http://www.isrctn.com/ISRCTN52132803). We started to recruit patients from 26 November 2018. At the original submission of the protocol, we have completed our pilot studies and have recruited five patients.

**Fig. 3:**
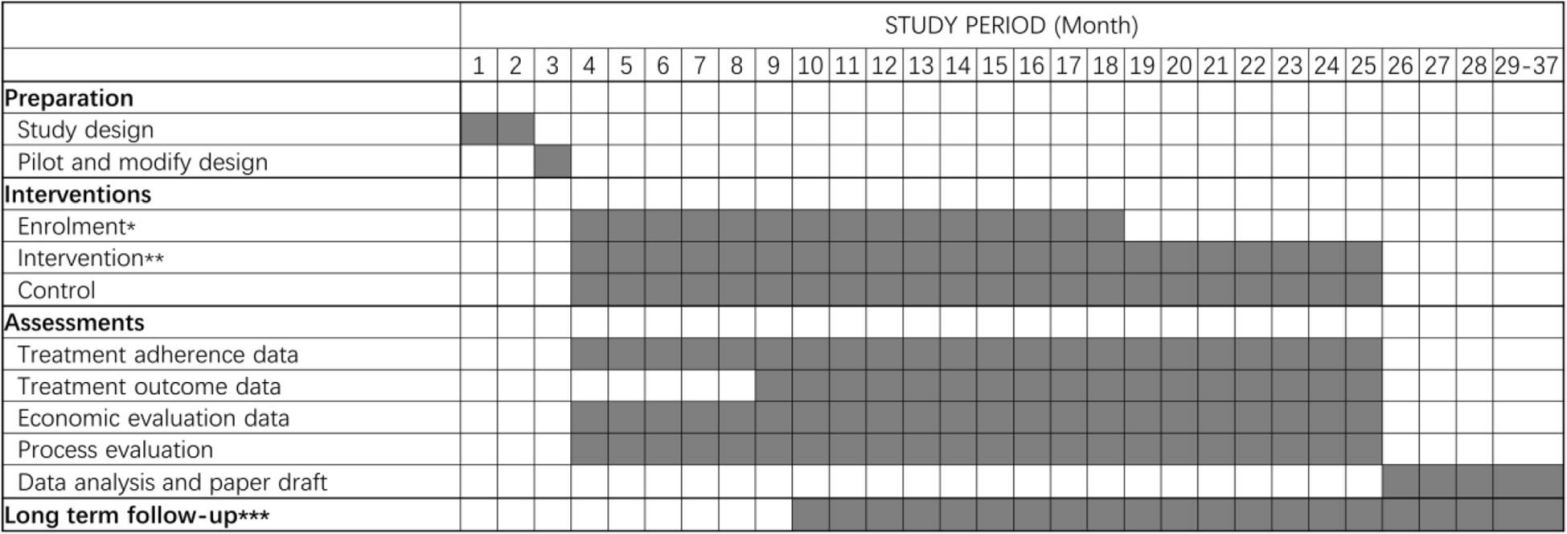
Study timeline. *Enrolment includes eligibility screen, informed consent, and allocation which will happened in 1 day for a patient. **Patients will be followed during their whole treatment period. ***Long-term follow-up will begin when a patient completes their treatment or they had a final adverse outcome including loss to follow-up, and will last for 12 months for each patient

The study will be done over a 28-month period, with a 3-month preparation and pilot phase, a 15-month patient recruitment phase, with a 6/7-month treatment phase for all patients, and a 3-month data analysis and write-up stage. We will disseminate trial results through research articles and policy briefs. We also plan to follow-up all participants 12 months after they end treatment using the TB reporting system to look at relapse and mortality rates between the treatment arms. Results of the follow-up study will be reported in a separate paper from the trial results. See Fig. 3 for details of the trial timeline.

## Additional file

Additional file 1: Standard Protocol items: Recommendations for Interventional Trials (SPIRIT) 2013 Checklist: recommended items to address in a clinical trial protocol and related documents. (DOC 125 kb)

## Abbreviations

CDC: Centre for Disease Control and Prevention
DMC: Data Management Committee
DOT: Directly observed treatment
E-monitor: Electronic monitor
FDC: Fix-dose combination
GEE: Generalised estimating equation
LMICs: Low-and middle-income countries
NTP: National Tuberculosis Programme
RCT: Randomised controlled trial
SAT: Self-administered therapy
SMS: Short Message Service
TB: Tuberculosis
TSC: Trial Steering Committee
UTAUT: Unified Theory of Acceptance and Use of Technology
VOT: Video-observed treatment
